# Characterizing oral microbial communities across dentition states and colonization niches

**DOI:** 10.1186/s40168-018-0443-2

**Published:** 2018-04-10

**Authors:** Matthew R. Mason, Stephanie Chambers, Shareef M. Dabdoub, Sarat Thikkurissy, Purnima S. Kumar

**Affiliations:** 1 Division of Periodontology, College of Dentistry, The Ohio State University, 4111 Postle Hall, 305, W 12th Avenue, Columbus, OH 43210 USA; 2 Present address: Division of Periodontology, University of North Carolina, Chapel Hill, NC USA; 3 Nationwide Children’s Hospital, Columbus, OH USA; 4 Present address: Great Beginnings Pediatric Dentistry, Asheville, NC USA; 5 Present address: Division of Pediatric Dentistry and Orthodontics, Cincinnati Children’s Hospital, Cincinnati, OH USA

**Keywords:** Bacteria, DNA, Pyrosequencing, 16S, Saliva, Supragingival, Subgingival, Acquisition

## Abstract

**Methods:**

The present study aimed to identify patterns and processes in acquisition of oral bacteria and to characterize the microbiota of different dentition states and habitats. Mucosal, salivary, supragingival, and subgingival biofilm samples were collected from orally and systemically healthy children and mother-child dyads in predentate, primary, mixed, and permanent dentitions. 16S rRNA gene sequences were compared to the Human Oral Microbiome Database (HOMD). Functional potential was inferred using PICRUSt.

**Results:**

Unweighted and weighted UniFrac distances were significantly smaller between each mother-predentate dyad than infant-unrelated female dyads. Predentate children shared a median of 85% of species-level operational taxonomic units (s-OTUs) and 100% of core s-OTUs with their mothers. Maternal smoking, but not gender, mode of delivery, feeding habits, or type of food discriminated between predentate microbial profiles. The primary dentition demonstrated expanded community membership, structure, and function when compared to the predentate stage, as well as significantly lower similarity between mother-child dyads. The primary dentition also included 85% of predentate core s-OTUs. Subsequent dentitions exhibited over 90% similarity to the primary dentition in phylogenetic and functional structure. Species from the predentate mucosa as well as new microbial assemblages were identified in the primary supragingival and subgingival microbiomes. All individuals shared 65% of species between supragingival and subgingival habitats; however, the salivary microbiome exhibited less than 35% similarity to either habitat.

**Conclusions:**

Within the limitations of a cross-sectional study design, we identified two definitive stages in oral bacterial colonization: an early predentate imprinting and a second wave with the eruption of primary teeth. Bacterial acquisition in the oral microbiome is influenced by the maternal microbiome. Personalization begins with the eruption of primary teeth; however, this is limited to phylogeny; functionally, individuals exhibit few differences, suggesting that microbial assembly may follow a defined schematic that is driven by the functional requirements of the ecosystem. This early microbiome forms the foundation upon which newer communities develop as more colonization niches emerge, and expansion of biodiversity is attributable to both introduction of new species and increase in abundance of predentate organisms.

**Electronic supplementary material:**

The online version of this article (10.1186/s40168-018-0443-2) contains supplementary material, which is available to authorized users.

## Background

Although the oral cavity is one of most microbe-rich environments in the human body, at birth, most children do not possess a colonized microbiome [1–3]. Upon exposure to the environment during and following the birthing process, colonization of the oral cavity occurs within 8–16 h [3]. Bacteria are introduced through a variety of vehicles, including diet, digit sucking, vertical transmission from parents, and horizontal transmission from caregivers and peers [4].

In the absence of natal teeth, the mucosal surfaces lining the oral cavity provide the only environment for bacterial colonization. The eruption of primary teeth creates two more niches for bacterial colonization, a supragingival habitat consisting of a non-shedding enamel tooth surface and a subgingival habitat composed of an abiotic tooth surface, the junctional epithelium and the epithelial lining of the gingival sulcus. Nearly 6 years following the establishment of this stable dentate environment, the primary dentition begins to exfoliate, giving way to the permanent dentition. This approximately 6-year phase when an individual has a mixture of teeth from both the primary and permanent dentitions is termed the mixed dentition, following which an adult permanent dentition is established. In order to understand the role of the microbiome in the predisposition to, or in prevention of, disease, it is essential to define the development of microbial assemblages in this ecosystem and the partitioning that occurs with the introduction of new colonization niches.

Development of the subgingival microbiome has been extensively studied in the context of two periodontal diseases that affect young children—aggressive periodontitis and puberty gingivitis [5–9]. Similarly, the salivary and supragingival microbial communities have been examined either in the context of a highly prevalent childhood disease—dental caries [10], or in relation to the role played by vertical and horizontal transmission in colonization [11, 12]. However, information regarding the development of a health-compatible oral microbiome is sparse and is gained largely from cultivation and targeted molecular approaches [13–17].

Our knowledge of the oral microbiome has expanded exponentially with the development of novel molecular methods that allow us to examine diversity, structure, function, and topography without the need to cultivate the individual components of the biofilm [18–20]. Recent investigations have revealed the existence of indigenous bacterial consortia that are specific to each body habitat and are ubiquitous among individuals (the core microbiome), implying that these species are uniquely adapted to the niche [21, 22], while emerging evidence indicates that there may be specific core microbiomes associated with distinct developmental states [23, 24]. This finding has significant relevance for the oral cavity, where eruption and shedding of teeth create distinct dentition development stages.

While a prospective follow-up of birth cohorts would be the ideal methodology to examine the time-course of oral bacterial colonization, longitudinal study designs suffer from issues associated with attrition of subjects due to (i) lack of commitment, (ii) geographic instability, (iii) disease onset, and (iv) lifestyle changes (e.g., children who begin smoking or vaping). Other challenges in longitudinal studies, especially those that span a period ranging from a few months of age to late adulthood, are maintaining uniformity of methods and quality control, staffing, and financing [25].

Therefore, the purpose of this investigation was to examine the patterns and processes involved in the development of the healthy oral microbiome and to trace the development of the subgingival and supragingival microbiomes by combining a cross-sectional clinical study with 16S rRNA gene sequencing and constraining comparisons to the core microbiome of each dentition cohort.

## Methods

Approval for this study was obtained from the Office of Responsible Research Practices at Nationwide Children’s Hospital (IRB07-00335) and at The Ohio State University (2015H0202) and carried out according to the approved guidelines. One hundred forty-three caries-free, systemically healthy children in between 1 day and 17 years of age were recruited, and demographic information, dietary, dental, and medical histories obtained following informed consent. An additional 60 caries-free children and their biological mothers were also recruited. The demographics and clinical characteristics of the subjects are shown in Additional file 1: Table S1. Subjects with chronic medical conditions, history of caries, antibiotic use within 3 months of study and those requiring antibiotics prior to dental treatment were excluded. Samples of saliva, oral mucosal biofilms (predentate), and supragingival and subgingival plaque were collected. The sample collection methodology is described in Additional file 2.

DNA was isolated; 16S rRNA gene libraries created and sequenced as described earlier [26]. The pipelines used for sequence analysis and statistical methods are described in Additional file 2.

## Results

### Acquisition of oral bacteria

#### The predentate community

**Fig. 1:**
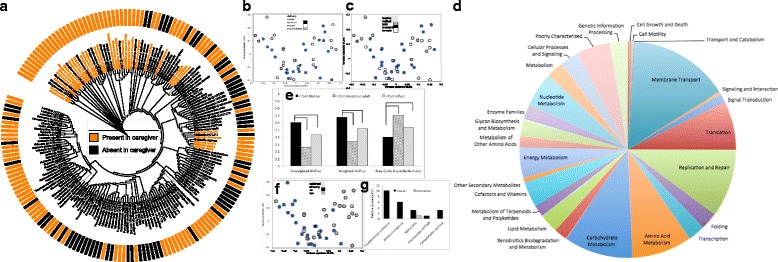
Bacterial acquisition in the predentate microbiome. Species-level operational taxonomic units (s-OTUs) that were identified in the predentate mucosa of 47 infants are shown in **a**. Bars indicate presence of species and are not sized by their abundances. Species that were part of the core predentate mucosal microbiome are indicated in orange font. Orange bars indicate s-OTUs that were shared by each child and their biological mother (based on 11 mother-child dyad samples). Principal component analysis of unweighted UniFrac distances colored by delivery mode (**b**) and feeding mode (**c**) are shown. The inferred functional potential encoded in the core predentate mucosal microbiome is shown in **d**. The pie slices are sized by relative abundances of genes encoding for each function. The similarity between each mother-child, infant-infant, and infant-unrelated adult dyad is shown in **e**. The metrics are unweighted UniFrac distances, weighted Unifrac, and Bray-Curtis similarity. Groups that are bracketed are significantly different from each other (*p* < 0.05, Dunn’s test). Principal component analysis of unweighted UniFrac distances of the predentate microbiome is shown in **f**. The samples are colored by maternal smoking (self reported). The relative abundances (expressed as a percent) of species that were significantly different between predentate infants of smokers and nonsmokers is shown in **g**

We examined acquisition of oral bacteria by first establishing a microbial catalogue from 1.4 million sequences from all 47 predentate mucosal samples. One hundred seventy-eight species-level operational taxonomic units (s-OTUs) belonging to 50 genera were identified (Fig. 1a), with 65 *±* 18 s-OTUs in each infant. The majority of these species were gram-positive facultatives, followed by gram-negative anaerobes. Only 33 out of the 178 species were shared by ≥ 75% of infants (core predentate microbiota, Fig. 1a), with 27 ± 5 core species in each infant. In each infant, these core species accounted for 45% (range of 23–61%) of the total microbiome. These core species belonged to the genera *Streptococcus*, *Gemella*, *Granulicatella*, and *Veillonella*, *s*imilar to previous reports on adult saliva [27–29]. PCoA of unweighted and weighted UniFrac distances did not evidence significant clustering based on mode of delivery (eutocic versus dystocic) or diet (breast-milk versus formula), (Fig. 1b, c).

Although the Human Microbiome Project and other studies promulgated a paradigm of a core microbiome consisting of a universal suite of species, emergent evidence, including work from our own group, points to the existence of a “core” set of functionalities within an ecosystem rather than species [30, 31]. Therefore, we used predictive metagenomic analysis (PICRUSt [32]) to estimate the functional roles of predentate bacteria. Eight hundred seventy-eight genes contributing to 26 functional pathways were identified. Importantly, 78% of the predicted functional potential (684 genes encoding for 22 functions) was shared by ≥ 75% of the infants. The predicted core functional endowments in this early microbiome related to membrane transport, carbohydrate metabolism, replication and repair, amino acid metabolism, translation, nucleotide metabolism, and energy metabolism, in decreasing order of abundance (Fig. 1d). Together, genes encoding for these functions accounted for 70% of the metagenome.

We then investigated if the maternal oral microbiome is a source of predentate bacteria. We first compared the salivary microbiota of 11 predentate infants with their biological mothers using both phylotype-based and branch-length-based measures. Unweighted and weighted UniFrac distances were significantly lower between each mother-infant dyad when compared to infant-unrelated female dyads (*p* < 0.05, Student *t* test, Fig. 1e), indicating that infants resembled their mothers in both community membership as well as structure even before the eruption of teeth and consequent addition of colonization niches. Each predentate child shared a median of 85% (range 38%–90%) of their s-OTUs with their mother, and all 33 core OTUs were present in the mothers (Fig. 1a). We then investigated if maternal habits affected the predentate dentition by correlating infant microbial profiles to self-reported maternal smoking. Out of the 47 predentate infants, those whose mothers were smokers demonstrated significant clustering as well as higher levels of *Fusobacterium nucleatum* and *Campylobacter concisus* (*p* < 0.05, FDR adjusted Wald test, DESeq2) when compared to those with nonsmoker mothers (Fig. 1f and g, and Additional file 1: Table S1).

#### Shifts in salivary signatures with addition of habitats

**Fig. 2:**
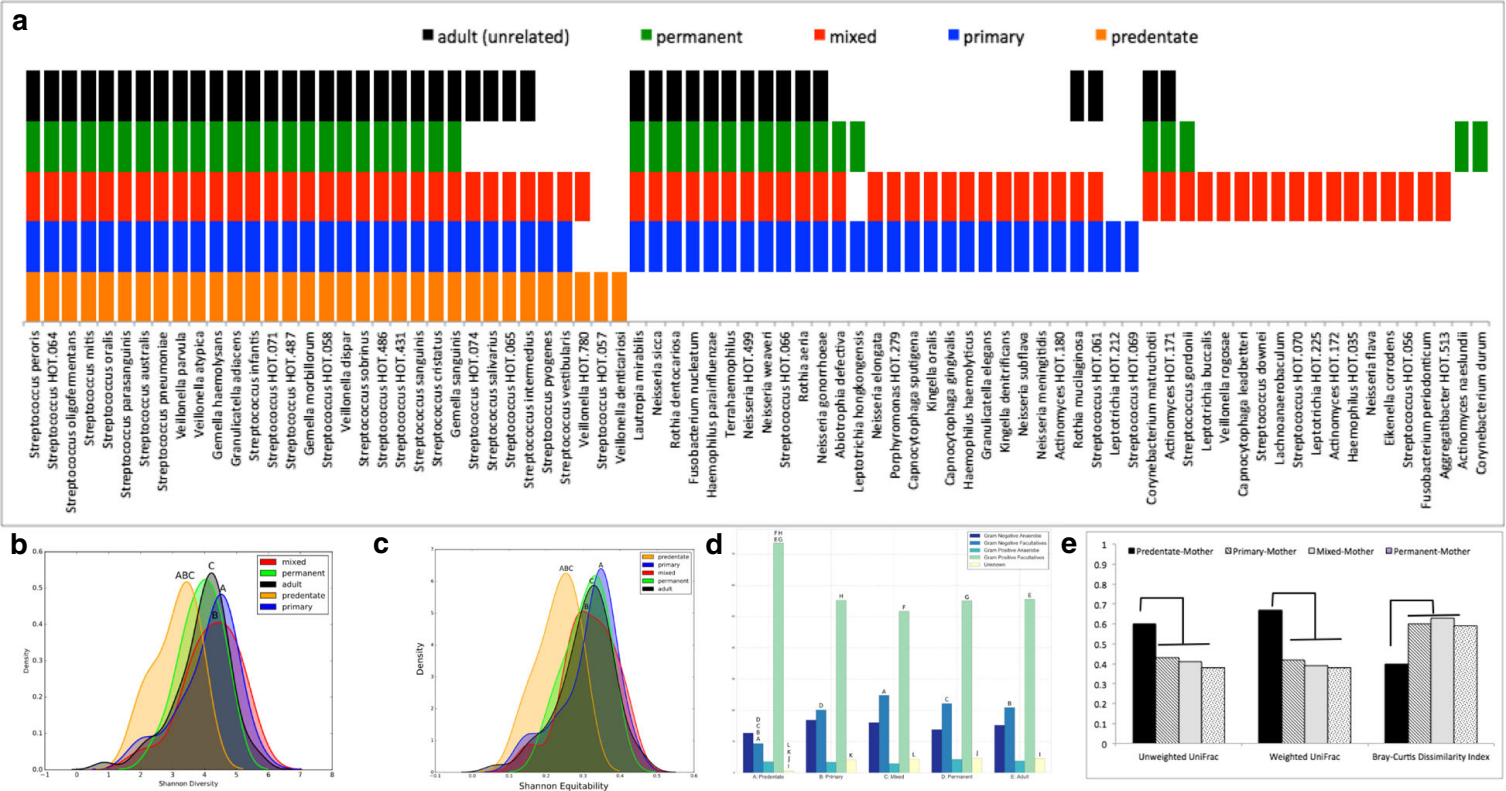
Phylogenetic shifts in the core salivary microbiome during various dentition states. **a** The salivary core microbiome (present in at least 75% of individuals in the group) of children in predentate, primary, mixed, and adult dentitions, as well as those of unrelated adults. Each bar represents presence of species. Data for **a** is presented in Additional file 4: Table S2. Shannon diversity index is shown in Fig. b and equitability in Fig. c. The distribution of species by gram staining characteristics and oxygen requirements in each dentition state is shown in Fig. d. Probable gram staining characteristics and oxygen requirements were attributed to uncultivated species based on phylogenetic relatedness to the closest cultivated species. In all the figures, groups that share the same symbol are significantly different from each other (*p* < 0.05, Dunn’s test). The similarity between children in different dentition states and their biological mother is shown in **e**. Groups that are bracketed are significantly different from each other (*p* < 0.05, Dunn’s test)

Comparisons of the salivary microbiota of 47 predentate, 59 primary, 47 mixed dentition, 50 permanent, and 60 adult dentitions revealed the existence of a very small, universal core community (consisting of 24 s-OTUs out of a total of 480) and larger dentition-specific cores (Fig. 2a). The primary salivary microbial community demonstrated a significant greater alpha diversity and equitability when compared to predentate infants, which was also observed in the mixed, permanent, and adult dentitions (*p* < 0.05, Dunn’s test) (Fig. 2b, c). This higher diversity was attributable to both community membership and structure, with 138 additional s-OTUs observed in the primary dentition (when compared to predentate infants), and successive additions of 32 species in the mixed dentition and 56 in the permanent, as well as increases in levels of species belonging to *Streptococcus*, *Gemella*, *Granulicatella*, and *Veillonella* (Additional file 2: Table S2). Predentate infants demonstrated significantly higher levels of gram-positive facultatives and lower levels of gram-negative facultatives than all dentate individuals (Fig. 2d). With the eruption of teeth, the similarity between the mother and child significantly decreased, and this divergence persisted through all dentition states (Fig. 2e). The introduction of primary teeth in the oral environment was accompanied by the largest expansion of functionality (1135 KEGG orthologs) between two dentition states (Additional file 3: Figure S1A and b and Additional file 4: Table S2). Of these 1135 KEGG orthologs, 4% (43 genes) were different between primary and mixed dentition states, and 1% was different in both the permanent (7 genes) and adult (14 genes) metagenomes. Thus, the expanded functional potential with the introduction of teeth into the oral cavity was also observed in a mature dentition. This was attributable to a 19% increase in genes encoding for membrane transport, 12% in carbohydrate metabolism genes, and 10% in amino acid metabolism (Additional file 3: Figure S1C). Cell motility and energy metabolism represented 7 and 6% of the increase respectively. In the permanent and adult dentitions, by contrast, 16% of the significantly enriched functional pathways belonged to membrane transport, with xenobiotic biodegradation and metabolism representing 13%, and cell motility and carbohydrate metabolism functions each representing 12% of the significant change (Additional file 3: Figure S1D).

#### Evidence for a temporally stable core salivary microbial community

At least 24 (range 24–33) core predentate s-OTUs were present in the core of the primary, mixed, permanent, or adult dentitions (Fig. 2a). In children with primary dentition, 28 additional s-OTUs (notably those belonging to the genera *Rothia*, *Fusobacterium*, *Actinomyces*, *Porphyromonas*, and *Capnocytophaga*) were identified in the salivary core, of which at least 11 s-OTUs (range 11–25) were also seen in the core of children with mixed and permanent dentition states. The mixed dentition core comprised of the largest number of s-OTUs (73), of which 55 were shared with the primary dentition. The permanent salivary core contained 42 total s-OTUs, 39 of which were shared with the mixed dentition. Thus, the data suggests that certain species acquired in the predentate and primary dentitions stably colonize the oral microbiome throughout life and may be the principal contributors of the core adult microbiome, a finding that warrants further evaluation using longitudinal studies.

### Habitat partitioning

#### Supragingival microbial profiles

**Fig. 3:**
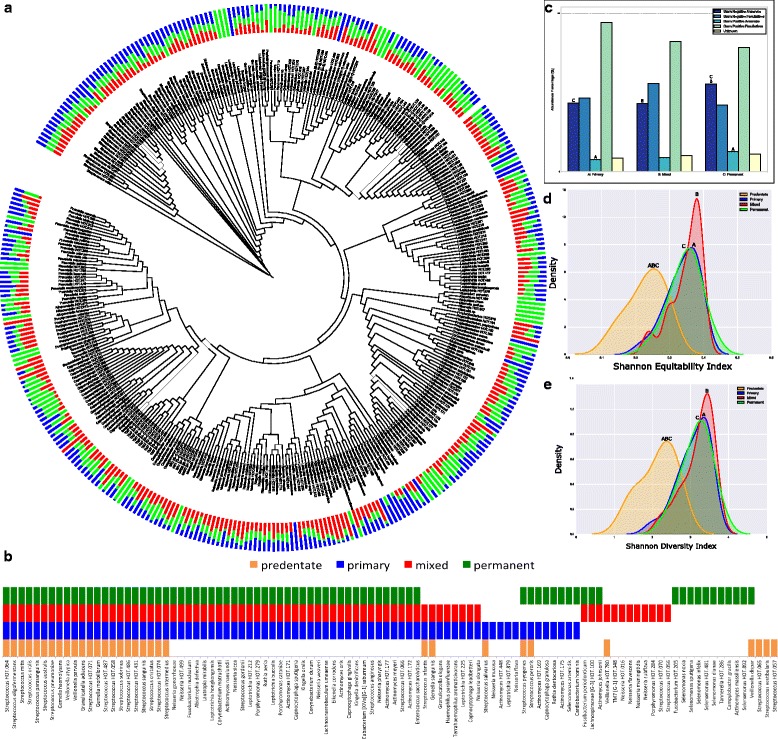
Phylogenetic characteristics of the supragingival microbiome. **a** A stacked bar chart of supragingival s-OTUs by dentition (phylogenetic tree constructed using the webserver iTOL.embl.de). Data for **a** is presented in Additional file 7: Table S3. The length of each bar represents the normalized mean relative abundance of a species. Data for this graph (**b**) shows the core supragingival microbiome in the three dentate stages. The bars represent species that were detected in 75% or more individuals in a group. The distribution of s-OTUs by gram staining characteristics and oxygen requirements in each dentition are shown in **c**, and Shannon diversity and equitability in **d** and **e** respectively. Probable gram staining characteristics and oxygen requirements were attributed to uncultivated species based on phylogenetic relatedness to the closest cultivated species. In all the figures, groups that share the same symbol are significantly different from each other (*p* < 0.05, Dunn’s test)

A total of 309 s-OTUs (132 *±* 31 in each child) were identified in the primary supragingival microbial community (Fig. 3a and Additional file 5: Table S4), of which 75 constituted the core (Fig. 3b). One hundred forty-eight of these 309 s-OTUs were also present in the predentate mucosal community. Three hundred eight s-OTUs (143 *±* 26 in each child) were identified in this habitat in the mixed dentition, of which 74 were part of the core, and 326 s-OTUs were identified in the permanent dentition (153 *±* 33 in each child), of which 77 formed the permanent core. Twenty-four out of the 75 species in the core primary community were also found in the core predentate mucosal flora, and 62 of these 75 species were identified in subsequent dentitions (Fig. 3b). Thus, approximately 50% of the s-OTUs identified in the supragingival habitat of any individual were shared by most individuals with the same dentition type, and 40% shared by all individuals irrespective of dentition state. The levels of anaerobic species (both gram positive and gram negative) were higher in permanent when compared to primary dentition (Fig. 3c). The primary dentition had significantly higher alpha diversity and equitability when compared to the predentate stage, with no differences between dentitions (Fig. 3d, e). The mixed dentition was compositionally intermediate between primary and permanent dentitions, but more similar to the primary dentition. The eruption of teeth was accompanied by a large functional increase (Additional file 6: Figure S2A), with acquisition of 2209 KEGG orthologs, representing 23 metabolic pathways (Additional file 6: Figure S2B and Additional file 7: Table S3). Only 33 additional genes were identified in the mixed dentition and 64 more in the permanent dentition. The higher functional potential with the emergence of teeth was primarily related to signal transduction, glycan biosynthesis and metabolism, cofactors and vitamins, replication and repair, cell motility, energy metabolism, amino acid metabolism, carbohydrate metabolism, and membrane transport (Additional file 6: Figure S2C and D).

#### Subgingival microbial profiles

**Fig. 4:**
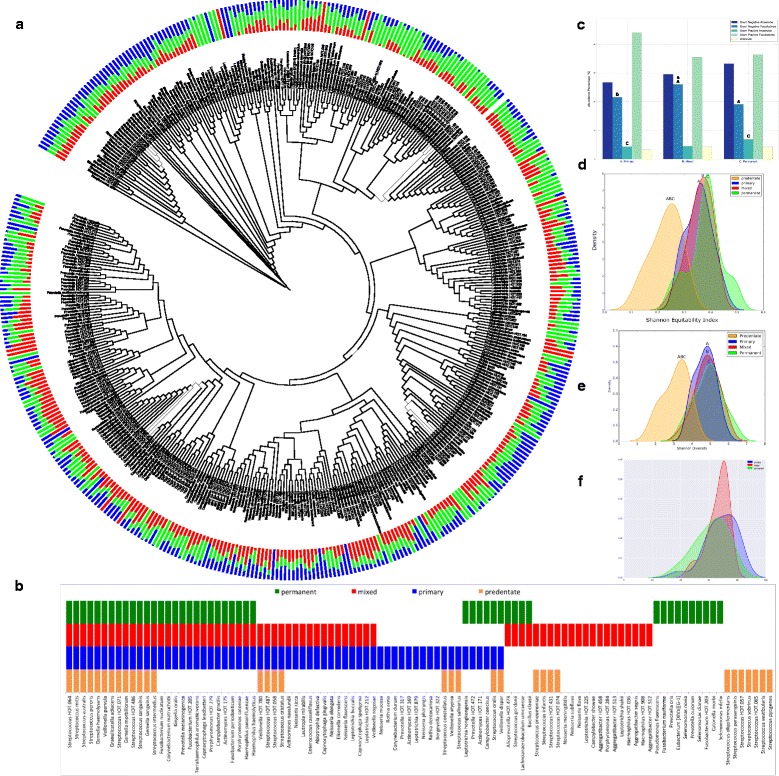
Phylogenetic characteristics of the subgingival microbiome. Figure 4A is a stacked bar chart representing the normalized mean relative abundances of subgingival s-OTUs by dentition (phylogenetic tree constructed using the webserver iTOL.embl.de). Data for Fig. 4a is presented in Additional File 9-supplemental table S5. **b** shows the core subgingival microbiome in the three dentate stages. The bars represent species that were detected in 75% or more individuals in a group. The distribution of s-OTUs by gram staining characteristics and oxygen requirements in each dentition are shown in panel **c**, and Shannon diversity and equitability in panels **d** and **e** respectively. Probable gram staining characteristics and oxygen requirements were attributed to uncultivated species based on phylogenetic relatedness to the closest cultivated species. Panel **f** shows the number of species shared between the supragingival and subgingival environments in all dentition states. In all the figures, groups that share the same symbol are significantly different from each other (*p* < 0.05, Dunn’s test)

A total of 365 s-OTUs belonging to 68 genera (152 *±* 35 in each child) were found in the primary subgingival community (Fig. 4a and Additional file 3: Table S4), of which 64 formed the core (Fig. 4b). One hundred sixty-seven of these 365 s-OTUs were also present in the predentate mucosal environment. Similarly, 378 s-OTUs (164 *±* 41 in each child) were identified in this habitat in the mixed dentition, of which 70 were part of the core; and 394 s-OTUs were identified in the permanent dentition (184 *±* 40 in each child) of which 47 formed the permanent core. Twenty-two out of the 64 species in the core primary subgingival community were also identified in the core predentate flora, and 50 of these 64 species in subsequent dentitions. Species present in all dentitions belonged to the genera *Streptococcus*, *Veillonella*, *Gemella*, *Granulicatella*, *Fusobacterium*, *Corynebacterium*, *Kingella*, *Prevotella*, *Terrahaemophilus*, *Capnocytophaga*, *Porphyromonas*, *Campylobacter*, *Actinomyces*, and *Haemophilus*. Thus, the subgingival biofilm demonstrated greater species richness as well as greater personalization in successive dentitions.

The mixed dentition contained significantly more gram-negative facultatives compared to all other dentition states, whereas the permanent dentition contained significantly more gram-positive anaerobes compared to the primary (Fig. 4c). There were no significant differences in diversity (Fig. 4d) and equitability (Fig. 4e) between dentition states; however, predentate mucosal diversity and equitability were both significantly lower than the other three dentitions (*p* < 0.05, Dunn’s test). Each individual demonstrated a median of 65% (ranging between 62% in permanent dentition and 69% in the primary) similarity between subgingival and supragingival habitats (Fig. 4f).

The introduction of primary teeth to the oral environment resulted in the largest influx (1791 KEGG-annotated functions) of genes in any dentition when compared to the predentate mucosa (Additional file 8: Figure S3A). Of these, 99% were carried on into both the mixed dentition (1779 genes) permanent (1778 genes) core metagenomes (Additional file 8: Figure S3B). Of the functional pathways gained with the introduction of teeth, 19% encoded for membrane transport, 12% were related to carbohydrate metabolism, and 9% to amino acid metabolism (Additional file 8: Figure S3C). Notably, cell motility and energy metabolism represented 5 and 7% of the increase respectively. After the final primary teeth exfoliated, 18% of the 310 significantly elevated functional pathways belonged to cell motility, 16% to membrane transport, 15% to carbohydrate metabolism, and 10% to amino acid metabolism (Additional file 8: Figure S3D).

## Discussion

To study acquisition of bacteria into the oral microbiome, we recruited 47 full-term, normal birthweight infants (between 1 day and 6 months of age) born to normal weight mothers with no history of systemic disease. This timeframe was deliberately selected since it allowed us to examine a cohort in whom the biological mother was not necessarily the sole caregiver and during a period of life when diet was largely milk-based with introduction of solids to some children and intentional antibiotic exposure had not yet occurred.

In the present investigation, infants shared 85% of their microbiota with their mothers, suggesting that the maternal oral microbiome plays a role in introducing microbial species to the child. While it is not within the scope of this study to definitively identify if this transmission is horizontal (shared utensils or saliva) or vertical, some elements of our data suggests that vertical (genetic) transmission might be a possible route of bacterial acquisition. One indication is that although all mothers carried *F*. *nucleatum* and *Campylobacter rectus*, only children of smokers carried these pathogens. We have previously identified high levels of these pathogens in the subgingival microbiomes of pregnant smokers [33], while other investigations have shown that these pathogens are capable of translocating to the placenta [34]. Emerging evidence also suggests that translocation of maternal oral bacteria to the placenta induces fetal immune tolerance, thereby promoting colonization with “familiar” species in the infant [35]. When we examine our data in this context, it brings up a scenario where high levels of these pathogens in the subgingival crevice might lead to placental translocation and subsequent fetal tolerance. Longitudinal investigations extending from pregnancy to post-partum are required to further investigate the hypothesis that the oral microbiome may be another “heritable” component of the human metagenome that is transferred from mother to offspring. Moreover, the majority of species shared by the infant and mother were also identified in all dentition states. Together, these two findings point to the powerful role the maternal oral microbiome and pre-natal environmental factors play in shaping the microbiome in the next generation.

“Vaginal seeding” is a practice based on evidence that passage through the birth canal initiates microbial colonization of the infant and is gaining momentum among mothers who give birth by Caesarian section [36]. Indeed, mode of delivery (vaginal versus C-section) has been shown to impact the oral microbiome [37]. In the present investigation, no differences could be discerned in the oral microbiomes of infants based on mode of delivery or feeding. While this could be due to the small sample size, evidence is emerging that maternal seeding of the fetal microbiome may commence in utero [34] and that the effect of delivery mode cannot be discerned after 6 weeks of life [38], lending credence to our findings.

It is known that the oral microbiome is highly personalized and that both host genotype and environmental factors dictate this personalization [18, 19, 39], yet when this idiosyncrasy becomes established is not fully understood. Our data suggest that bacterial acquisition follows a biological blueprint and that microbial fingerprints are developed at the predentate stage. However, predictive functional modeling indicates that although infants may be phylogenetically idiosyncratic, they are colonized by functionally equivalent species, a trait that was also seen in the dentate cohorts. This corroborates recent studies describing the microbiome as taxonomically heterogeneous yet functionally congruent [30, 31] and signifies that each individual is colonized by bacterial consortia that are well adapted to his/her ecosystem requirements.

The primary dentition is a stage of greatest expansion in phylogenetic and functional diversity in the oral microbiome. While this could be attributed to either change in diet or increased colonization niches, our data provide strong evidence that change in habitat plays a major role. The first line of evidence is that our predentate and primary dentition cohorts overlapped to some degree in ages, and a diet questionnaire revealed that several of the predentate children had been introduced to solid (“adult”) foods. The microbiome did not cluster based on “adult” versus “infant” food. The second line of evidence comes from the metagenomic inferences. The functionalities acquired with the introduction of teeth into the oral cavity relate to adhesion, cellular multiplication, and biofilm formation, notably, membrane transport, cell motility, type IV secretion, oxidative phosphorylation, chemotaxis, and flagella assembly, indicating that the functional requirements of the new habitat drive the types of species that colonize this niche. The mixed dentition phase appears to be a stage of greatest transition, with several novel yet functionally equivalent species trying to gain a foothold with the eruption of new teeth.

The development of the subgingival biofilm mimicked the same general trends of the development of the salivary core across dentition states. Twenty-two of the 33 species found in the predentate mucosal microbiome were also seen in the primary subgingival community, highlighting the foundational role the predentate microbiome (and, by extension, the maternal microbiome) plays in the development of the periodontal microbial community. An increase in cell motility was notable with the exfoliation of all primary teeth, possibly due to the developing spatial characteristics of the subgingival pocket.

## Conclusions

In summary, within the limitations of a cross-sectional study design, the following conclusions may be drawn: bacterial acquisition in the oral microbiome is influenced by maternal microbiota. However, personalization begins very early in life, with the eruption of primary teeth. This personalization is limited to phylogeny; functionally, individuals exhibit few differences, indicating that microbial assembly follows a defined schematic that is driven by the functional requirements of the ecosystem. This early microbiome forms the foundation upon which newer communities develop as more colonization niches emerge, and expansion of biodiversity is attributable to both introduction of new species and abundance of predentate organisms. Further inquiry utilizing longitudinal prospective cohorts will serve to establish the role that initial microbial colonization plays in long-term microbiome development.

## Additional files

Additional file 1: **Table S1.** Demographic and clinical data of subjects participating in study. (XLSX 59 kb)

Additional file 2: Methods used in the study. (DOCX 172 kb)

Additional file 3: **Supplemental Figure S1:** Functional shifts in the core salivary microbiome during various dentition states. Figure 1a shows a Bland-Altman plot of changes in the relative abundance of core functional genes in the salivary microbiome between the different dentitions. Each point is a functional gene. Points above and below the red median line represent genes demonstrating higher abundances in the specified dentition. Red points indicate genes with significantly different abundances (*p* < 0.05, FDR adjusted Wald test). Figure 1b shows the number of functional genes that were different between two subsequent dentitions. For example, the bar “Primary” represents genes that were different between predentate and primary dentitions, while “Mixed” represents number of genes differing between primary and mixed dentitions. Figures 1c and d represent the functions encoded by these genes in the primary and permanent dentitions respectively. (PDF 541 kb)

Additional file 4: **Table S2.** Relative abundances of species level operational taxonomic units (s-OTUs) in the salivary microbiome. Values are represented as a percent with standard deviations. (XLSX 73 kb)

Additional file 5: **Table S4.** Relative abundances of species level operational taxonomic units (s-OTUs) in the supragingival microbiome. Values are represented as a percent with standard deviations. (XLSX 62 kb)

Additional file 6: **Supplemental Figure S2:** Functional shifts in the core supragingival microbiome during various dentition states. Figure 2a shows a Bland-Altman plot of changes in the relative abundances of core functional genes in the supragingival microbiome between the different dentitions. Each point is a functional gene. Points above and below the red median line represent genes demonstrating higher abundances in the specified dentition. Red points indicate genes with significantly different abundances (*p* < 0.05, FDR adjusted Wald test). Figure 2b shows the number of functional genes that were different between two subsequent dentitions. For example, the bar “Primary” represents genes that were different between predentate and primary dentitions, while “Mixed” represents number of genes differing between primary and mixed dentitions. Figures 2c and d represent the functions encoded by these genes in the primary and permanent dentitions respectively. (PDF 428 kb)

Additional file 7: **Table S3.** Predicted functions in the salivary, supragingival and subgingival microbiomes. Values are represented as a percent abundance. (XLSX 45 kb)

Additional file 8: **Supplemental Figure S3:** Functional shifts in the core subgingival microbiome during various dentition states. Figure 3a shows a Bland-Altman plot of changes in the relative abundances of core functional genes in the subgingival microbiome between the different dentitions. Each point is a functional gene. Points above and below the red median line represent genes demonstrating higher abundances in the specified dentition. Red points indicate genes with significantly different abundances (*p* < 0.05, FDR adjusted Wald test). Figure 3B shows the number of functional genes that were different between two subsequent dentitions. For example, the bar “Primary” represents genes that were different between predentate and primary dentitions, while “Mixed” represents number of genes differing between primary and mixed dentitions. Figures 3c and d represent the functions encoded by these genes in the primary and permanent dentitions respectively. (PDF 511 kb)

Additional file 9: **Table S5.** Relative abundances of species level operational taxonomic units (s-OTUs) in the subgingival microbiome. Values are represented as a percent with standard deviations. (XLSX 73 kb)
