# Supplementary material for: Characterizing oral microbial communities across dentition states and colonization niches
Source: Microbiome. 2018 Apr 10;6:67. doi: 10.1186/s40168-018-0443-2 (PMC5891995; doi:10.1186/s40168-018-0443-2)
Supplement: Supplementary file 2 — Methods used in the study. (DOCX 172 kb) [file 40168_2018_443_MOESM2_ESM.docx]

METHODS

**Consent and recruitment:**

Approval for this study was obtained from the Office of Responsible Research Practices at Nationwide Children’s Hospital and The Ohio State University (IRB07-00335 and 2015H0202 respectively). 143 caries-free, systemically healthy children in between 1 day and 17 years of age were recruited, and demographic information, dietary, dental and medical histories obtained following informed consent. An additional 60 caries-free children and their biological mothers were also recruited.

**Sample collection**

Saliva was obtained by placing a single toothette underneath the tongues of the child and mother for 30 seconds. For predentate children, a mucosal sample was collected by wiping a toothette along the buccal mucosa and alveolar ridges. From dentate children and all mothers, supragingival plaque was collected from the facial surfaces of anterior teeth using a sterile swab (Microbrush®, Grafton, WI, USA) Subgingival plaque was obtained by placing endodontic paper points (Dentsply Caulk, Milford, DE, USA) in the sulci of anterior teeth. All samples were stored at -200C until processing. A single calibrated investigator obtained the samples and data.

**DNA isolation**:

200μl of phosphate buffered saline (PBS) was added to 50μl of saliva, paper points (subgingival samples) and microbrushes (supragingival samples). All samples were vortexed for one minute, and DNA isolated using a Qiagen MiniAmp kit (Valencia, CA) according to manufacturer’s “tissue” protocol, quantified using Qubit fluorometer and stored at -200C.50ng of DNA was used generate libraries using the Nextera DNA Sample preparation kit (Illumina) following the manufacturer's user guide. The initial concentration of DNA was evaluated using the Qubit® dsDNA HS Assay Kit (Life Technologies). Two regions of the 16S rRNA genes were sequenced: V1–V3 (spanning E.coli 16S gene regions 8-27 and 519-536) and V7–V9 (spanning E.coli 16S gene regions 1099-1114 and 1528-1541). The primers used for sequencing have been previously described(Kumar et al., 2011). Briefly, a single step PCR with 22 cycles of amplification was used to amplify the 16S rRNA genes as well as to introduce adaptor sequences and sample-specific bar-code oligonucleotide tags into the DNA.The 16s amplicons were quantified using the Quant-iT PicoGreen dsDNA reagent and kit (Invitrogen). Equimolar concentrations of each amplicon were pooled and multiplexed bacterial tag-encoded FLX amplicon pyrosequencing was performed using the Titanium platform (Roche Applied Science, Indianapolis, IN, USA) as previously described (Kumar et al., 2011). Samples were randomized across sequencing runs to prevent batch effects. Positive controls consisting of a pre-mixed consortium of lab strains, as well as negative control (elution buffer), were included in each run.

Two primers were used, since each primer is capable of detecting a range of genera that the other fails to recover. Together they allow the recovery of a wider range of the microbiome than is possible with a single primer alone. However, some genera are picked up by both primers. Thus, to prevent overcounting, the number of sequences assigned to an OTU by both primers was reduced by half. Primer averaging was carried out as previously described(Kumar et al., 2011) using the implementation in the PhyloTOAST software suite (Dabdoub et al., 2016). Analyses were conducted using the QIIME(Caporaso et al., 2010) and PhyloToAST(Dabdoub et al., 2016) pipelines. The sequences were binned by sample and quality checked (QIIME split_libraries.py) by requiring sequences to have an average quality score of 30 over a sliding window of 50bp and length >200 bp. Chimeric sequences were depleted using UCHIME (v. 6.1, identify_chimeric_seqs.py). Taxonomic identity was established by alignment to the HOMD database(Chen et al., 2010) using BLAST v2.2.22 as required by QIIME.

An average of 39,231 sequences were generated per sample (range 14,241 to 40,738). The predentate group demonstrated the lowest number of sequences (14241 to 31,431). Alpha (within-group) and beta (between-group) diversity were computed. Since emerging evidence does not support rarefying the microbiome to compensate for sequencing effort(McMurdie and Holmes, 2014), we used linear regression models to correct for sequencing depth (phyloseq in R). The Shannon and Abundance Coverage Estimator (ACE) metrics were used as estimators of alpha diversity. Both phylogenetic (UniFrac and weighted UniFrac) and non-phylogenetic (Bray–Curtis) distance matrices were utilized to estimate beta diversity. Significance of group-wise clustering under beta diversity metrics was interrogated using Adonis with 999 permutations. Principal Coordinates Analysis (PCoA, QIIME principle_coordinates.py) and Linear Discriminant Analysis (LDA, scikit-learn v0.18.1) were performed for dimensionality reduction of the beta diversity distance matrices. The resulting coordinate data was visualized using PhyloTOAST (PCoA.py and LDA.py). Phylogenetic trees were created using PyNAST and align_seqs.py with QIIME. The resulting tree files along with the OTU abundance counts were processed with PhyloTOAST (iTol.py) to calculate normalized mean relative abundance values for each OTU per group and replace the database OTU IDs with readable short taxonomic names for visualization with the iTOL(Letunic and Bork, 2011) (<http://itol.embl.de/>, version 3.4.1) web application.

A core microbiome was computed for each dentition state to include all s-OTUs present in at least 75% of the subjects in a dentition group. The functional potential of each microbial community was estimated using PICRUSt (Langille et al., 2013). A core functional potential was computed for each dentition state to include all KEGG (Kyoto Encyclopedia of Genes and Genomes)(Kanehisa and Goto, 2000) entries present in at least 75% of the subjects in a dentition group. Comparisons of functional potential between clinical groups were made in the context of the KEGG ontological hierarchies and statistical analysis of differential functional potential was performed using R and DESeq2(Love et al., 2014).

Probable gram staining characteristics and oxygen requirements were attributed to uncultivated species based on phylogenetic relatedness to the closest cultivated species.

Chen T, Yu WH, Izard J, Baranova OV, Lakshmanan A, Dewhirst FE (2010). The human oral microbiome database: A web accessible resource for investigating oral microbe taxonomic and genomic information. Database : the journal of biological databases and curation 2010(baq013.

Dabdoub SM, Fellows ML, Paropkari AD, Mason MR, Huja SS, Tsigarida AA, Kumar PS (2016). Phylotoast: Bioinformatics tools for species-level analysis and visualization of complex microbial datasets. Scientific reports 6(29123.

Kanehisa M, Goto S (2000). Kegg: Kyoto encyclopedia of genes and genomes. Nucleic Acids Res 28(1)*:*27-30.

Kumar PS, Brooker MR, Dowd SE, Camerlengo T (2011). Target region selection is a critical determinant of community fingerprints generated by 16s pyrosequencing. PLoS One 6(6)*:*e20956.

Langille MG, Zaneveld J, Caporaso JG, McDonald D, Knights D, Reyes JA, Clemente JC, Burkepile DE, Vega Thurber RL, Knight R *et al.* (2013). Predictive functional profiling of microbial communities using 16s rrna marker gene sequences. Nature biotechnology 31(9)*:*814-821.

Letunic I, Bork P (2011). Interactive tree of life v2: Online annotation and display of phylogenetic trees made easy. Nucleic Acids Res 39(Web Server issue)*:*W475-478.

Love MI, Huber W, Anders S (2014). Moderated estimation of fold change and dispersion for rna-seq data with deseq2. Genome Biol 15(12)*:*550.

McMurdie PJ, Holmes S (2014). Waste not, want not: Why rarefying microbiome data is inadmissible. PLOS Computational Biology 10(4)*:*e1003531.
