# Supplementary material for: Characterizing oral microbial communities across dentition states and colonization niches
Source: Microbiome. 2018 Apr 10;6:67. doi: 10.1186/s40168-018-0443-2 (PMC5891995; doi:10.1186/s40168-018-0443-2)
Supplement: Supplementary file 6 — Supplemental Figure S2: Functional shifts in the core supragingival microbiome during various dentition states. Figure 2a shows a Bland-Altman plot of changes in the relative abundances of core functional genes in the supragingival microbiome between the different dentitions. Each point is a functional gene. Points above and below the red median line represent genes demonstrating higher abundances in the specified dentition. Red points indicate genes with significantly different abundances (p < 0.05, FDR adjusted Wald test). Figure 2b shows the number of functional genes that were different between two subsequent dentitions. For example, the bar “Primary” represents genes that were different between predentate and primary dentitions, while “Mixed” represents number of genes differing between primary and mixed dentitions. Figures 2c and d represent the functions encoded by these genes in the primary and permanent dentitions respectively. (PDF 428 kb) [file 40168_2018_443_MOESM6_ESM.pdf]

Supplemental Figure 2

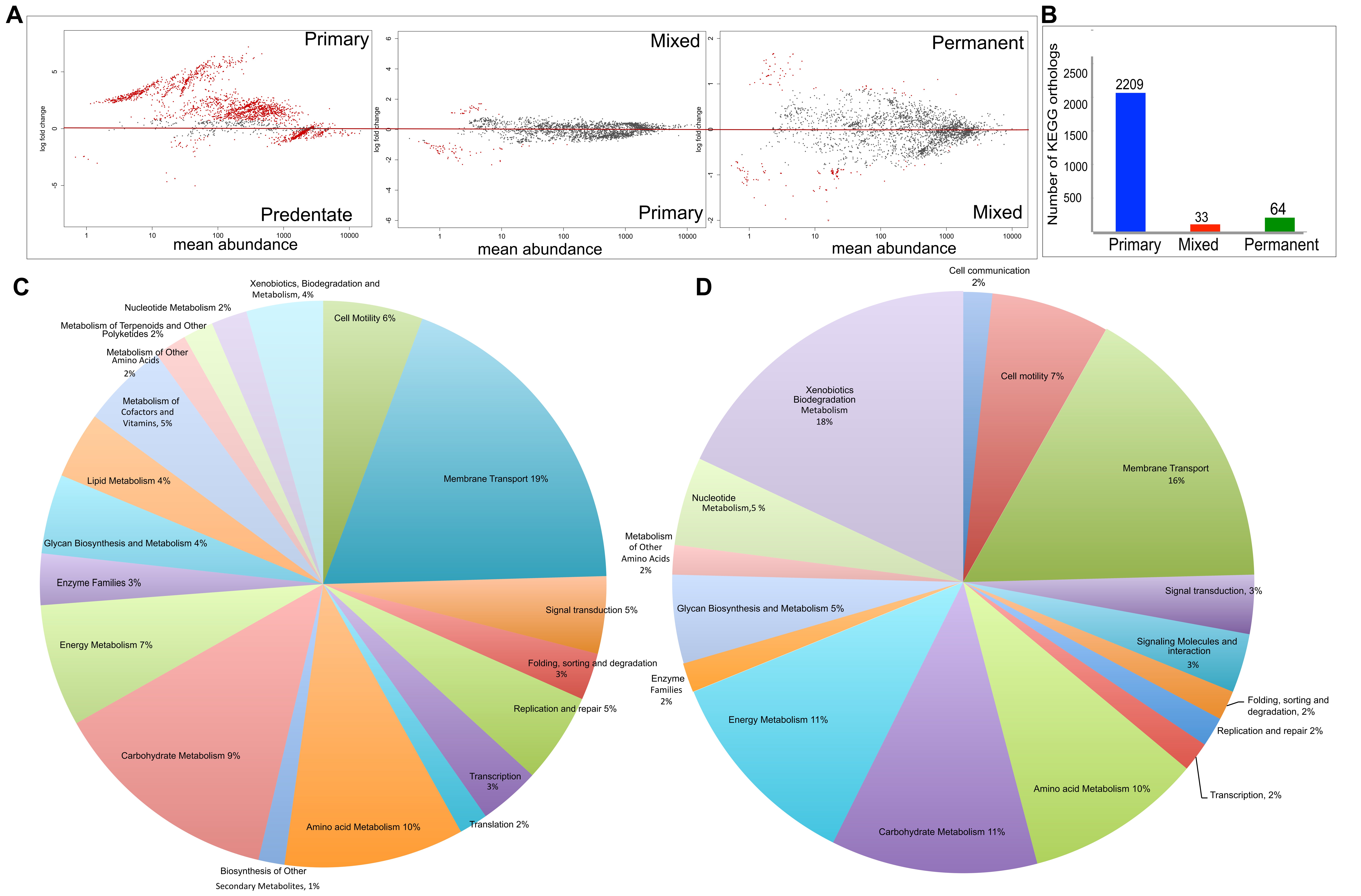

Functional shifts in the core supragingival microbiome during various dentition states. Figure 2A shows a Bland-Altman plot of changes in the relative abundances of core functional genes in the supragingival microbiome between the different dentitions. Each point is a functional gene. Points above and below the red median line represent genes demonstrating higher abundances in the specified dentition. Red points indicate genes with significantly different abundances ($p<0.05$, FDR adjusted Wald test). Figure 2B shows the number of functional genes that were different between two subsequent dentitions. For example, the bar "Primary" represents genes that were different between predentate and primary dentitions, while "Mixed" represents number of genes differing between primary and mixed dentitions. Figures 2C and 2D represent the functions encoded by these genes in the primary and permanent dentitions respectively.
